# Anterior reduction and C1-ring osteosynthesis with Jefferson-fracture reduction plate (JeRP) via transoral approach for unstable atlas fractures

**DOI:** 10.1186/s12891-021-04628-4

**Published:** 2021-08-30

**Authors:** Qiang Tu, Hu Chen, Zhan Li, Yuyue Chen, Aihong Xu, Changrong Zhu, Xianhua Huang, Xiangyang Ma, Jianhua Wang, Kai Zhang, Qingshui Yin, Jianzhong Xu, Hong Xia

**Affiliations:** 1 Department of Orthopaedics, PLA General Hospital of Southern Theatre Command: People’s Liberation Army General Hospital of Southern Theatre Command, Guangzhou, 510010 Guangdong China; 2 Department of Orthopaedics, Southwest Hospital, Third Military Medical University, Chongqing, 400038 China; 3 The First School of Clinical Medicine, Southern Medical University, Guangzhou, 510010 Guangdong China; 4 Guangzhou University of Chinese Medicine, Guangzhou, 510006 Guangdong China

**Keywords:** Atlas, Fractures, Transoral approach, Surgery, Osteosynthesis

## Abstract

**Background:**

To introduce a novel transoral instrumentation in the treatment of unstable fractures of the atlas.

**Methods:**

From January 2008 to May 2018, 22 patients with unstable C1 fractures who received Jefferson-fracture reduction plate (JeRP) via transoral approach were retrospectively analyzed. The case history and the radiographs before and after surgery were noted. The type of fracture, the reduction of the fracture, and position of the internal fixation were assessed through preoperative and postoperative CT scans.

**Results:**

All 22 patients successfully underwent anterior C1-ring osteosynthesis using the JeRP system, with a follow-up of 26.84 ± 9.23 months. Among them, 9 patients had transverse atlantal ligament (TAL) injury, including 3 in Dickman type I and 6 in type II. The preoperative lateral mass displacement (LMD) decreased from 7.13 ± 1.46 mm to 1.02 ± 0.65 mm after the operation. Bone union was achieved in all patients without implant failure or loss of reduction. There were no surgery-related complications, such as wound infection, neurological deficit, or vertebral artery injury. However, atlantoaxial dislocation occurred in 3 patients with Dickman type I TAL injury 3 months postoperatively without any neurological symptoms or neck pain.

**Conclusions:**

Transoral C1-ring osteosynthesis with JeRP is an effective surgical strategy to treat unstable atlas fractures with a safe, direct, and satisfactory reduction. The primary indication for the JeRP system is an unstable fracture (Gehweiler type I/III) or/ and TAL injury (Dickman type II).

**Supplementary Information:**

The online version contains supplementary material available at 10.1186/s12891-021-04628-4.

## Background

The atlas has a unique ring structure with no vertebral body or spinous processes, which provides more flexibility and a greater range of motion (ROM) than other vertebrae of the spine [1]. As a transitional structure, it is an indispensable part of the craniovertebral junction, allowing the axial loading to be transferred from the occiput to the axis. The junctions of the lateral mass connected to both the anterior and posterior arches are relatively thinner, making them the weakest points of C1 and more susceptible to fracture [2]. That’s why fractures with two or more breaks commonly occur in the C1-ring [3]. In general, the types of atlas fracture can be described as stable and unstable based on the integrity of transverse atlantal ligament (TAL) and adjacent vertebrae. Unstable C1 fractures are characterized by the high-grade transverse spread of lateral mass on an open-mouth radiograph.

To date, posterior atlantoaxial and occipitocervical fusion techniques have been widely used in the treatment of unstable C1 fractures and have achieved satisfactory results [4]. However, posterior fusion techniques decrease the ROM of the craniocervical junction. Regarding this problem, the anterior C1-ring osteosynthesis is an appropriate option to safely and effectively perform ORIF via the transoral approach [5]. However, surgeons preferred the posterior C1-ring osteosynthesis to avoid surgical site infection, even if this technique cannot completely reduce and stabilize C1 anterior arch fractures. In 2004, Ruf [6] first reported a C1-C2 rotation function-preserving technique of C1 osteosynthesis via transoral approach, which not only enabled an anatomic reconstruction of the anterior arch of C1 but also achieved bony union. The study by Ma [2] demonstrated that the anterior technique of C1-ring ORIF with direct manipulation at the lateral masses was safe and effective for the treatment of highly unstable cases. However, the internal fixation device used in C1-ring osteosynthesis is not specialized for atlas fractures, and it is unlikely to achieve near anatomical reduction for C1 fracture directly and safely, particularly in the case of C1 anterior arch fractures. To our knowledge, there is no report on specialized apparatus for C1 osteosynthesis using a transoral approach.

In this study, we described a novel Jefferson-fracture reduction plate (JeRP) system (Wego Corporation, China) via the transoral approach. It is originally designed and clinically applied to treat unstable atlas fractures to preserve the motion function of the atlantoaxial joint.

## Methods

This research program was approved by the institutional review committee of People’s Liberation Army (PLA) General Hospital of Southern Theatre Command. Each patient signed a medical informed consent document preoperatively.

**Fig. 1:**
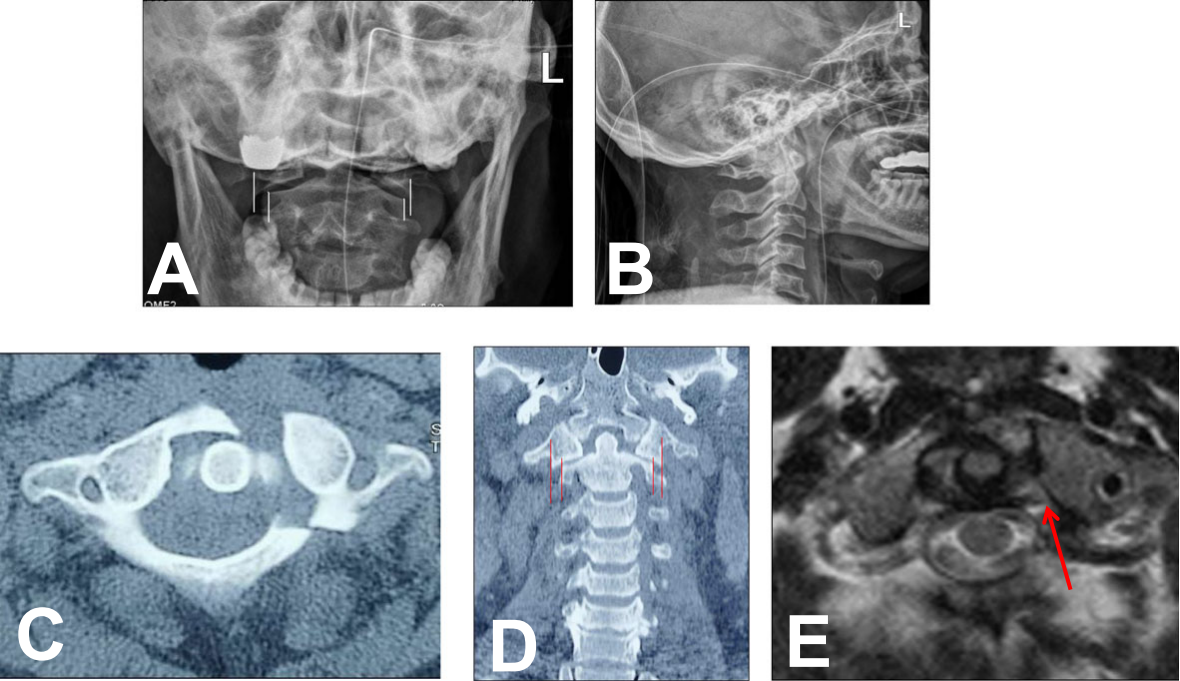
A represent case (**A**, **B**, **C**, and **D**) Preoperative X-ray and CT showed C1 fracture. **C** There were fracture lines in both anterior and posterior arches (Gehweiler type III). The LMD value measured on the open X-ray film was 7.28 mm (**A**), and the value measured on the coronal CT film was 6.54 mm (**D**). The MRI showed that the TAL had a high signal (red arrow) at the attachment point of the left lateral mass, considering an avulsion injury (Dickman type II). While compression of the spinal cord was not observed (**E**)

**Fig. 2:**
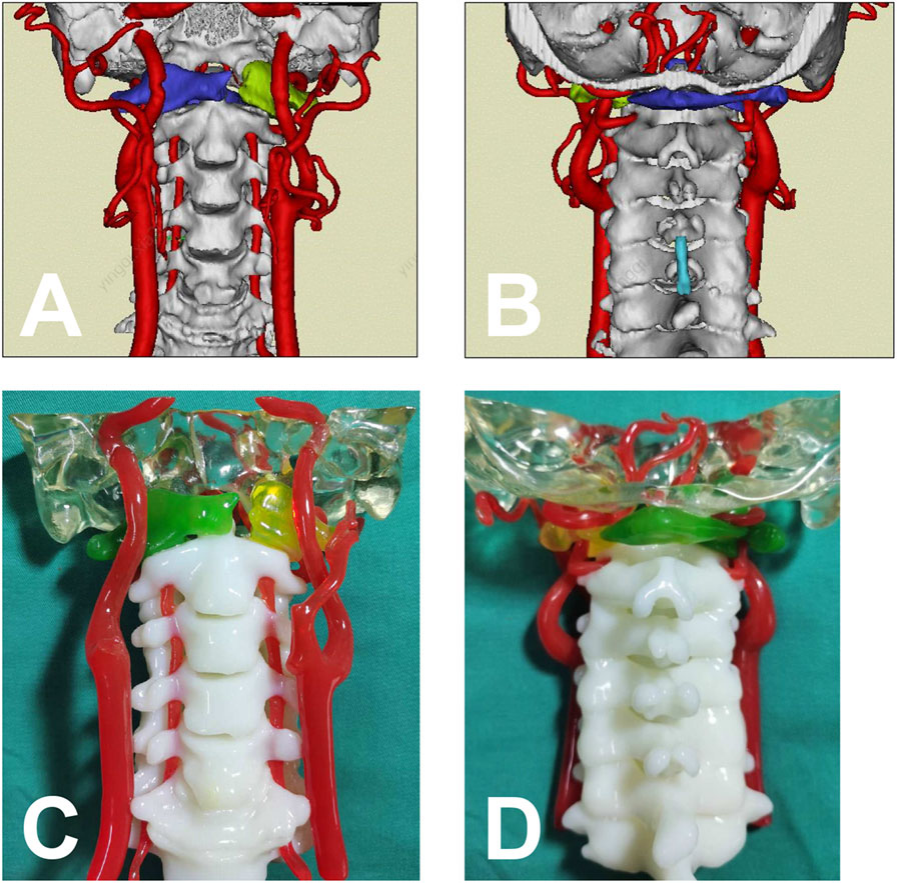
3D reconstruction of anatomical digital model (**A** and **B**) and 1:1 scale 3D printed model of the cervical spine (**C** and **D**). The model showed that the fracture line of C1 was located at the junction of the left lateral mass and the anterior and posterior arches, and the fractured end (green and yellow, respectively) was shifted outwards (**C** and **D**)

From Jan 2008 to May 2018, 22 patients with unstable C1 fractures who received JeRP via transoral approach were retrospectively analyzed. Patients with fractures of the adjacent vertebrae of the axis or occipital condyle were excluded. All patients suffered neck pain, stiffness, and limited range of motion in the neck. None of them had neurological deficit. On admission, all patients underwent preoperative x-ray film, CT scans, and MRI (Figs. 1 and 2). The lateral mass displacement (LMD) of the atlas was measured from the coronal reconstructed view of the CT scans. The TAL integrity was evaluated by preoperative MRI. All patients underwent 4-6 kg skull traction preoperatively and intraoperatively in the hope that the displaced lateral mass might close at least partially.

### Design of the JeRP system

**Fig. 3:**
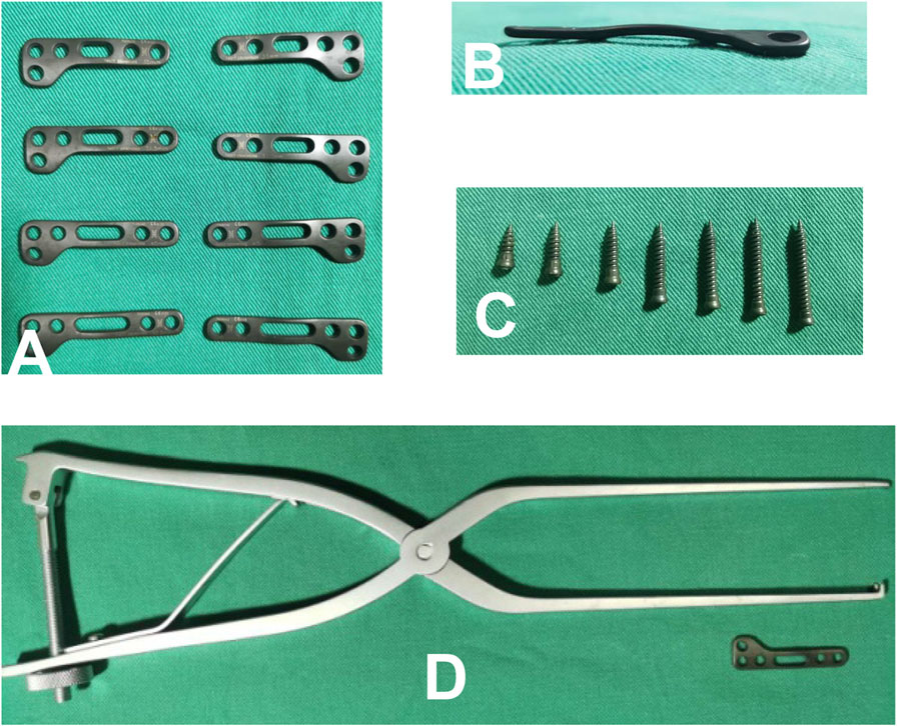
Different views of the JeRP system. Top and side views of titanium alloy plate (**A** and **B**). There are two types of L-shaped plates, left and right, corresponding to C1 fractures with the fracture line closed to the left or right lateral mass (**A**). The plate has a slight arc, fitting the physiological curvature of C1 (**B**). Different sizes of self-tapping screws (**C**) and the JeRP specialized repositor were shown (**D**)

**Fig. 4:**
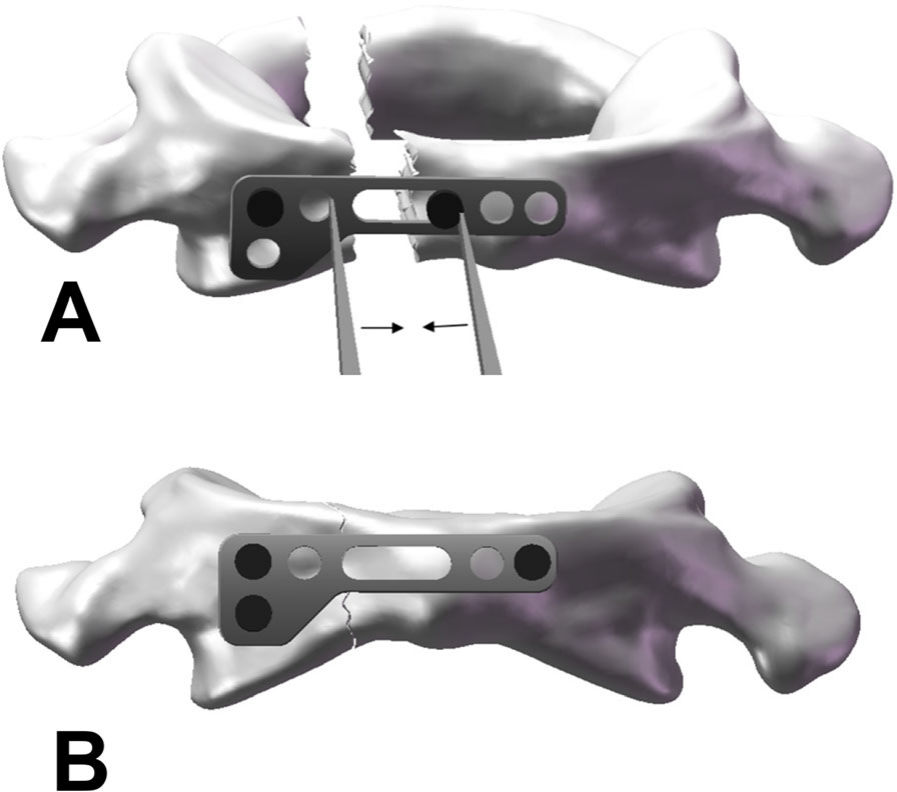
Schematic diagram of JeRP plate treating C1 fracture. **A** The JeRP plate was placed in the center of the anterior arch of C1 and screwed into the upper screw hole on the lateral mass near the fracture line. A temporary reduction screw was screwed into the long-elliptic hole, and the nut was about 3 mm away from the bone surface of the anterior arch. The JeRP specialized repositor was used to compress the plate and the temporary screw. **B** After making the fracture end fit tightly, the other lateral mass screws were tightened, the temporary screw was removed, and the entire reduction fixation process was completed

The JeRP system consists of titanium alloy plates, self-tapping screws, specialized repositor, and corresponding surgical instruments (Fig. 3). The JeRP plates shape like an “L”, which is consistent with the anatomical morphology in front of the atlas, and the lateral mass end of it is provided with two circular fixing holes fixed to the lateral mass of C1. On the side views, the plate has a slight arc, fitting the physiological curvature of C1. Additionally, there are two types of L-shaped plates, left and right, corresponding to C1 fractures with the fracture line closed to the left or right lateral mass respectively. The middle of the system is a long-elliptic hole in which a temporary reduction screw can slide. Notably, with the help of the specialized repository, the compression force can be gently applied to pull together the fractured ends for reduction. The working principle of the JeRP system in the treatment of C1 fracture is shown in Fig. 4.

### Surgical technique

**Fig. 5:**
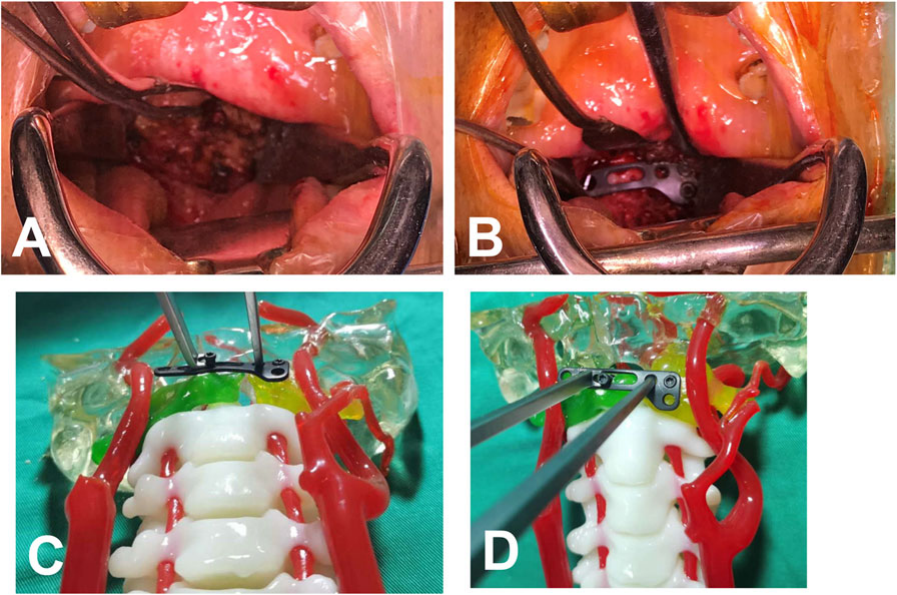
Surgical Technique of JeRP system. Soft tissue was dissected to fully expose the surgical field, including the anterior arch, fragment bone, and lateral mass of the atlas (**A**). The JeRP plate is placed in the anterior aspect of atlas (**B**). Preoperative surgery simulation with 3D printed solid model (**C**, **D**)

All surgeries were performed by senior spine surgeons. Under general anesthesia with transnasal endotracheal intubation, the patients were placed in a supine position with skull traction weighing 4-6 kg. Codman oral retractor was used to expose the oropharynx. A 3-4 cm longitudinal midline incision along the posterior pharyngeal wall was made, and the soft tissue was dissected to fully expose the surgical field, including the anterior arch, fragment bone, and lateral mass of atlas (Fig. 5A). The ideal entry point, the trajectory of the screw, and the length of the JeRP plates were determined based on the preoperative CT measurement. The anterior tubercle of C1 was polished with a high-speed drill. After removing the soft tissue between bony fragments, the JeRP plate was placed in the anterior aspect of the atlas (Fig. 5B). By palpating the medial edge and lower margin of the lateral mass, we could determine the optimal entry point of the screws. First, one lateral mass screw close to the fracture line was installed and tightened to stabilize the plate. Then, a temporary reduction screw was placed in the anterior arch of C1, which could be moved in the runner of the plate, and the nut was about 3 mm away from the bone surface of the anterior arch. The specialized repositor was installed between a temporary screw and the complex comprising the lateral mass and the plate (Fig. 5C, D). The compression force was gently applied via the specialized repositor to pull together the fractured ends and make them fit tightly. Next, the other lateral mass screws were tightened, the temporary screw was removed, and the entire reduction fixation process was completed. Finally, the JeRP plate was affixed to the anterior arch, with the intraoperative fluoroscopy confirming that the reduction of fracture and position of internal fixation were satisfying. After completing the JeRP implant procedure, the wound was soaked with povidone-iodine for 15 min and then was closed in the muscular and mucosal layers respectively with interrupted sutures.

### Postoperative treatment

**Fig. 6:**
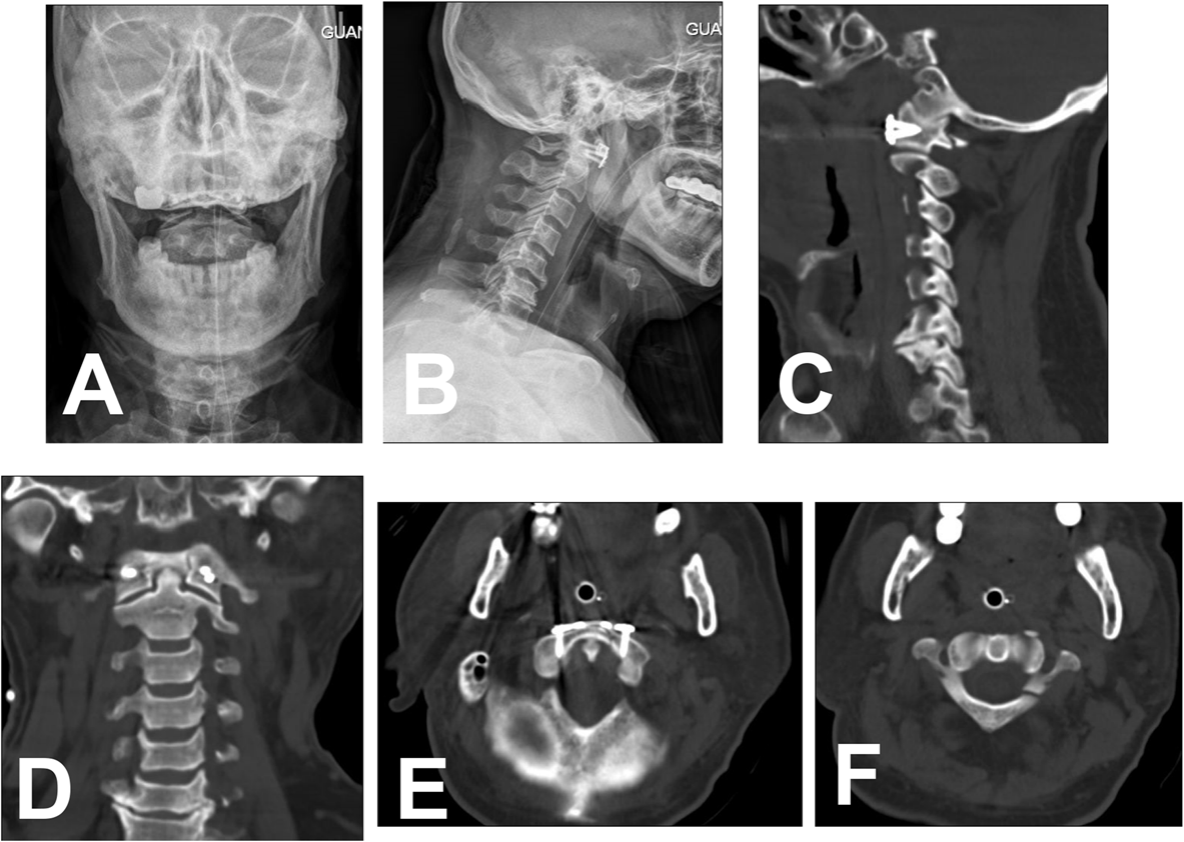
Postoperative X-ray and CT showed a good position of the JeRP plate (**A**, **B**, **C**, **D**, and **E**). CT scan showed that the preoperative LMD decreased from 6.54 mm to 0 mm after the surgery (**D**), and C1 fracture was anatomically reduced (**E** and **F**)

The transnasal endotracheal catheter was maintained for 2 days after the operation, and a nasogastric feeding tube was kept for 1 week. The fluid diet was started after the removal of the nasogastric tube and the normal diet was resumed 3 weeks postoperatively. All patients were given 5- to 7-day intravenous antibiotics as preventive interventions after surgery and kept a neck collar for 3 months. Postoperative CT was used to assess the efficacy of the C1 reduction and the accuracy of the screw placement. What’s more, CT scans and X-ray film of the cervical spine were performed at the third, sixth, and twelfth months after surgery to evaluate bony healing, reduction, and stability of the C1–C2 (Fig. 6). At the final follow-up, the atlanto-dens interval (ADI) was calculated based on flexion-extension radiographs. Furthermore, a visual analogue scale (VAS) was performed to evaluate the degree of neck pain.

## Results

The demographic data of patients were listed in Table 1. The incision of the posterior pharyngeal wall in all patients was well-healed without any infection or dehiscence. No vertebral artery or spinal cord injury occurred during the operation. According to Gehweiler’s classification system, 7 of 22 patients presented with type I fractures, and 15 of 22 patients had type III fractures. The C1-rings were separated into various number of fragments, including 2 separate fragments (in 7 patients), 3 fragments (in 13 patients), and 4 fragments (in 2 patients). Nine patients had confirmed TAL injury (3 had type I and the rest had type II based on Dickman’s classification), while the other 13 patients had no or unconfirmed TAL injury. The surgical and clinical outcomes were listed in Tables 2 and 3. All operations were completed successfully without surgery-related complications, such as wound infection, neurological deficit, or vertebral artery injury. The preoperative LMD decreased from 7.13 ± 1.46 mm to 1.02 ± 0.65 mm after the operation. Bone union was achieved in all patients without implant failure or loss of reduction. At the final follow-up, the mean VAS score was 0.28 ± 0.13 points. Moreover, the upper cervical range of motion in all patients was well preserved. However, 3 patients had an ADI of more than 4 mm at the last follow-up without any neurological symptoms or neck pain. One patient had limited cervical movement due to the penetration of the atlanto-occipital facet with lateral mass screws but didn’t receive any special treatment because it didn’t cause any obvious pain.

**Table 1:** Demographic Data

Variables	N
Sex (Male,n)	22 (12)
Age (years)	32 ~ 67
Follow-up time (months)	26.84 ± 10.23
Type of fractures (n)	
Type I	7
Type III	15
Other types	0
TAL injury	
Type I	3
Type II	6
Uncertain	13

**Table 2:** Surgical outcomes

Variables	
Surgical time (min)	181.32 ± 185.00
Blood loss (ml)	144.21 ± 155.67
Hospital stay(d)	19.00 ± 10.55
Complications	1 patient had limited cervical movement due to the penetration of the atlanto-occipital facet with lateral mass screws, but no obvious pain and no special treatment. No surgery-related complications,such as wound infection, neurological deficit, or vertebral artery injury.

**Table 3:** Clinical outcomes

Parameters	Preoperative	Postoperative	Follow-up	*P*
LMD (mm)	7.13 ± 1.46	1.02 ± 0.65	0.53 ± 0.21	<0.0001
VAS	7.42 ± 3.92	2.17 ± 1.33	0.28 ± 0.13	<0.0001
Complications	3 patients with Dickman type I TAL injury occured atlantoaxial dislocation 3 months postoperatively without any neurological symptoms or neck pain			

## Discussion

Most stable atlas fractures can be treated conservatively [7]. As for unstable C1 fractures, surgical strategies have become the standard of care. Unstable atlas fracture is usually accompanied by TAL injury, presenting as separation of the lateral masses, and subluxation or dislocation of atlantoaxial joint [5]. There is also a point that it was atlas fractures, instead of C1 posterior arch fractures, that destroyed the stability of the upper cervical spine. The displacement of bone fragments or atlantoaxial dislocation puts the spinal cord in danger and can result in severe complications like paraplegia or even death [8, 9]. The goal of the treatment is to reduce the fracture, correct dislocated fracture, stabilize the atlantoaxial joint, and preserve the maximum ROM of the upper cervical spine [10, 11]. However, the surgical strategies for unstable C1 fractures are still controversial.

### Value of C1-ring osteosynthesis in unstable atlas fracture

Conservative therapy in unstable atlas fracture for several months may lead to severe discomfort and a high incidence of bony nonunion [12]. Simultaneously, the inconsistency and mechanical instability of the occipitocervical junction may restrict motion and cause persistent neck pain [13]. To date, the posterior atlantoaxial or occipitocervical fusion is supposed to be the main surgical method, but it will result in limited range of motion of the upper cervical spine. The ideal treatment method is limited fixation without restricting the ROM of the upper cervical spine [14]. For this, many spine surgeons gave up the posterior fusion and preferred C1-ring osteosynthesis [15, 16]. The question regarding the relationship between C1-ring osteosynthesis and the integrity of the TAL is still controversial. Traditionally, the integrity of TAL is the key to determining the stability of C1 fractures. The rule of Spence [17] showed that total LMD over 6.9 mm on open-mouth radiographs correlated with rupture to TAL had important clinical value in determining whether surgical intervention was needed, but this is currently being questioned. What’s more, other tissues can help maintain C1-C2 stability and restrict motion despite rupture of TAL. Some scholars’ findings have shown that the significance of axial ligamentous tension of craniocervical junction has been underrated [18]. Because of its unique anatomical structure and biomechanical environment, the occipitocervical junction is mainly stabilized by the ligamentous complex of C0-C1-C2 [13]. Previous literature has shown that C1 burst fractures are axial load injury for which the integrity of secondary stabilizers comprising the alar ligaments, facet capsule, and neck musculature is easier to maintain. C1-ring osteosynthesis techniques can restore the axial tension of the ligamentous complex of C0-C1-C2 through the reduction of the fracture. Studies have shown that even with the rupture of TAL, C1-ring osteosynthesis can provide sufficient stability under physiological load. Thus, incompetence of TAL may not be a contraindication to C1-ring osteosynthesis. The conventional definition of C1 instability based on the integrity of TAL underestimates the number of fractures requiring surgical intervention and overestimates the number of fractures requiring C1-C2 fusion.

### Advantage of anterior C1-ring osteosynthesis using JeRP system

C1-ring osteosynthesis using both posterior and transoral approaches has been published [18]. With posterior C1-ring osteosynthesis techniques, posterior arch fracture and the lateral mass displacement could be satisfactorily reduced by the compression force on the end of bilateral mass screws. However, it makes the front of the lateral mass screw swing laterally, leading to insufficient reduction of anterior arch fracture of C1 [19]. In addition, complications of this approach are related to the technical difficulty of obtaining a direct reduction and potential for residual postoperative stiffness relating to the approach. In all, with limited retrospective data, it is difficult to verify the effectiveness of C1 osteosynthesis between posterior and transoral approaches without comparative studies and larger cohort size.

Anterior direct reduction of the atlas fractures promotes the rate of bony union of fractures by improving the integrity of the C0-C1-C2 complex structure. This approach has a good safety profile, avoiding the fusion of important motion segments while restoring the C0-C2 height. Results of transoral C1-ring osteosynthesis for unstable atlas fractures have been verified over the last decade. However, many surgeons are hesitant about this technique because of unfamiliarity with the transoral approach, the theoretically increased risk of infection. The universal shortcoming of transoral C1 osteosynthesis published previously is that reduction of C1 fracture is only an acceptable repair rather than anatomical reconstruction. The transoral C1-ring osteosynthesis is technically challenging, and there are no specialized devices or spinal implants designed for treating unstable atlas fractures. The main problem with current techniques is that the posterior pharyngeal soft tissue is not thick enough to cover the plate or rod, thus increasing the risk of wound complications. Additionally, it is difficult to implement a satisfactory reduction of atlas fractures in deep and narrow space. Meanwhile, the end of the lateral mass screw via transoral approach is too high, which is likely to result in the crack of the wound of the posterior pharyngeal wall or postoperative dysphagia. In this study, the JeRP system is introduced for anterior C1-ring osteosynthesis. The advantage of this system lies in the use of a dedicated reduction instrument, which can not only satisfy the reduction of the fracture end but also place the fixation screws in the fracture reduction state without being affected by the reduction instrument. It is generally acceptable to follow the principle that the screws don’t penetrate the edge of the lateral mass into the atlanto-occipital joint or enter the atlantoaxial joint. The lateral mass is wedge-shaped, with higher external and lower internal structures. The screw has an internally deviated insertion point, allowing the screw to easily enter the joint. In our paper, 1 lateral mass screw was observed to enter the atlanto-occipital joint. In the series, 22 patients had bone fusion without any wound infection and dehiscence being observed. The main advantage of the JeRP system is that it can ideally reduce the C1 fracture via the anterior approach and the inserted plate and screws will not interfere with the midline wound closure. The inconsistency of the C0-C1 and C1-C2 joints is rectified, and the ligamentous tension band of the craniocervical junction is regained as well. As far as we know, this new technology can minimize lateral mass displacement. The JeRP system appears to be a safe and effective method to deal with unstable C1 fractures. It can achieve ideal bone fusion and preserve the range of motion of the craniocervical junction.

The JeRP system was designed for treating unstable C1 fractures without affecting the TAL, and its indication is very narrow. In the actual process, we also applied the JeRP system to C1 fractures with TAL rupture, and achieved satisfactory results. Among them, 3 patients with Dickman type I TAL injury suffered atlantoaxial dislocation postoperatively, while the patients with Dickman type II TAL injury had good effect. Hence, the primary indication for the JeRP system is an unstable C1 fracture (Gehweiler type I/III) with or without TAL injury (Dickman type II).

### Limitations

Limitations of this paper include the lack of quantified range for preserving the motion of atlantooccipital and atlantoaxial joints, small sample size, retrospective design, and the possibility of selection bias.

## Conclusions

Transoral C1-ring osteosynthesis with the JeRP system is an effective option for treating unstable C1 fractures and can achieve safe, direct, and satisfactory reduction. Even if the TAL is ruptured, the atlantoaxial joint can remain relatively stable. TAL injury may not necessarily be an absolute contraindication for ORIF for atlas fracture. However, further studies are needed to investigate the long-term effect of atlantoaxial instability.

## Supplementary Information

**Additional file 1.**

## Data Availability

The datasets generated during and analyzed during the current study are not publicly available due to them containing information that could compromise research participant privacy/consent, but are available from the corresponding author on reasonable request.

## Abbreviations

JeRP: Jefferson-fracture reduction plate
LMD: Lateral mass displacement
TAL: Transverse atlantal ligament
ADI: Atlanto-dens interval
VAS: Visual analogue scale

## References

[1] Wang J, Zhou Y, Zhang ZF (2012). Direct repair of displaced anterior arch fracture of the atlas under microendoscopy: experience with seven patients. Eur Spine J.

[2] Ma WH, Xu NJ, Hu Y (2013). Unstable atlas fracture treatment by anterior plate C1-ring osteosynthesis using a transoral approach. Eur Spine J.

[3] Zou XB, Ouyang BP, Wang BB (2020). Motion-preserving treatment of unstable atlas fracture: transoral anterior C1-ring osteosynthesis using a laminoplasty plate. BMC Musculoskelet Disord.

[4] Laubach M, Pishnamaz M, Scholz M, et al. Interobserver reliability of the Gehweiler classification and treatment strategies of isolated atlas fractures: an internet-based multicenter survey among spine surgeons. Eur J Trauma Emerg Surg. 2020. 10.1007/s00068-020-01494-y. 10.1007/s00068-020-01494-y PMC8825399 32918554

[5] Guo W, Lin Y, Huang JW (2020). Treatment strategy of unstable atlas fracture: a retrospective study of 21 patients. Medicine (Baltimore).

[6] Ruf M, Melcher R, Harms J (2004). Transoral reduction and osteosynthesis C1 as a function-preserving option in the treatment of unstable Jefferson fractures. Spine.

[7] Bednar DA, Almansoori KA (2016). Solitary C1 posterior fixation for unstable isolated atlas fractures: case report and systematic review of the literature. Glob Spine J.

[8] Fiedler N, Spiegl UJA, Jarvers JS (2020). Epidemiology and management of atlas fractures. Eur Spine J.

[9] Schleicher P, Scholz M, Kandziora F (2019). Recommendations for the diagnostic testing and therapy of atlas fractures. Z Orthop Unfall.

[10] Kandziora F, Scholz M, Pingel A (2018). Treatment of atlas fractures: recommendations of the spine section of the German Society for Orthopaedics and Trauma (DGOU). Global Spine J.

[11] Smith RM, Bhandutia AK, Jauregui JJ (2018). Atlas fractures: diagnosis, current treatment recommendations, and implications for elderly patients. Clin Spine Surg.

[12] Zhao ZS, Wu GW, Lin J (2019). Management of Combined Atlas Fracture with type II odontoid fracture: a review of 21 cases. Indian J Orthopaed.

[13] Zhang YS, Zhang JX, Yang QG (2018). Posterior osteosynthesis with monoaxial lateral mass screw-rod system for unstable C1 burst fractures. Spine J.

[14] Hu Y, Xu RM, Albert TJ (2014). Function-preserving reduction and fixation of unstable Jefferson fractures using a C1 posterior limited construct. J Spinal Disord Tech.

[15] Ottenbacher A, Bettag M (2017). Resolution of traumatic vertebral artery dissection and occlusion after repositioning and posterior C1-ring osteosynthesis of a displaced Jefferson burst fracture. Acta Neurochir.

[16] Koller H, Resch HM (2010). A biomechanical rationale for C1-ring osteosynthesis as treatment for displaced Jefferson burst fractures with incompetency of the transverse atlantal ligament. Eur Spine J.

[17] Woods RO, Inceoglu S, Akpolat YT (2018). C1 lateral mass displacement and transverse Atlantal ligament failure in Jefferson's fracture: a biomechanical study of the "rule of Spence". Neurosurgery.

[18] Shatsky J, Bellabarba C, Nguyen Q (2016). A retrospective review of fixation of C1 ring fractures--does the transverse atlantal ligament (TAL) really matter?. Spine J.

[19] Kandziora F, Chapman JR, Vaccaro AR, et al. Atlas fractures and atlas osteosynthesis: a comprehensive narrative review. J Orthop Trauma. 2017;31 Suppl 4:S81–S89. 10.1097/BOT.0000000000000942 28816879

